# Characterization and Clinical Significance of Natural Variability in Hepatitis B Virus Reverse Transcriptase in Treatment-Naive Chinese Patients by Sanger Sequencing and Next-Generation Sequencing

**DOI:** 10.1128/JCM.00119-19

**Published:** 2019-07-26

**Authors:** Ya Fu, Yongbin Zeng, Tianbin Chen, Huijuan Chen, Ni Lin, Jinpiao Lin, Xiaofeng Liu, Er Huang, Songhang Wu, Shu Wu, Siyi Xu, Long Wang, Qishui Ou

**Affiliations:** a First Clinical College, Fujian Medical University, Fuzhou, China; b Department of Laboratory Medicine, The First Affiliated Hospital of Fujian Medical University, Fuzhou, China; Memorial Sloan Kettering Cancer Center

**Keywords:** characterization, HBV, next-generation sequencing, reverse transcriptase, Sanger sequencing, significance, treatment-naive, variability

## Abstract

Mutations in hepatitis B virus (HBV) reverse transcriptase (RT) are associated with nucleos(t)ide analogue (NA) resistance during long-term antiviral treatment. However, the characterization of mutations in HBV RT in untreated patients has not yet been well illustrated. The objective of this study was to investigate the characterization and clinical significance of natural variability in HBV RT in treatment-naive patients. HBV RT sequences were analyzed in 427 patients by Sanger sequencing and in 66 patients by next-generation sequencing. Primary or secondary NA resistance (NA^r^) mutations were not found, except A181T in RT (rtA181T) by Sanger sequencing, but they were detected by next-generation sequencing. Mutations were found in 56 RT amino acid (aa) sites by Sanger sequencing, 36 of which had mutations that could lead to changes in B or T cell epitopes in the RT or S protein. The distribution of mutations was diverse in different sections within the RT region. Multiple mutations showed significant association with HBV DNA, HBsAg, HBeAg, age, and severity of liver fibrosis. Mutations at rt251, rt266, rt274, rt280, rt283, rt284, and rt286 were found most in the advanced liver disease (ALD) group by next-generation sequencing. The present study demonstrates that next-generation sequencing (NGS) was more suitable than Sanger sequencing to monitor NA^r^ mutations at a low rate in the treatment-naive patients, and that mutations in the RT region might be involved in the progression to ALD.

## Abstract (precis)

Mutations in hepatitis B virus (HBV) reverse transcriptase (RT) are associated with nucleos(t)ide analogue (NA) resistance during long-term antiviral treatment. However, the characterization of mutations in HBV RT in untreated patients has not yet been well illustrated. The objective of this study was to investigate the characterization and clinical significance of natural variability in HBV RT in treatment-naive patients.

## INTRODUCTION

Hepatitis B virus (HBV) is the most common cause of acute and chronic liver disease in China. Patients infected by HBV during infancy or early childhood are likely to develop into chronic hepatitis B (CHB) and have an increased risk of progression to liver cirrhosis (LC) and hepatocellular carcinoma (HCC) (1). HBV is an enveloped partially double-stranded DNA virus with an approximately 3.2-kb genome encoding four open reading frames (ORFs), pre-S/S, pre-C/C, HBX, and polymerase. The polymerase gene consists of four domains, as follows: the terminal protein, spacer, RNase H, and reverse transcriptase (RT). The HBV genome is replicated through RT using pregenomic RNA (pgRNA) as the template. However, RT lacks proofreading capacity in the process of viral replication, resulting in various genomic variants. Under the selective pressure of antiviral agents and the host immune defense, the fittest variants tend to survive (2).

Nucleos(t)ide analogue (NA) resistance is closely associated with mutations in the RT region. For instance, M204V/I in RT (rtM204V/I) is a classical lamivudine (LMV) resistance mutation, which also greatly reduces susceptibility to telbivudine (LdT); rtA181T/V is not only reported as an adefovir dipivoxil (ADV) resistance mutation, but rtA181T/V also confers a decreased susceptibility to LMV, LdT, tenofovir disoproxil fumarate (TDF), and tenofovir alafenamide (TAF); rtM204V/I and rtL180M together with rtI169T, rtV173L, rtM250V, rtT184G, or S202I/G can lead to resistance to entecavir (ETV) (3). However, since the S gene completely overlaps the RT region, the mutations in the RT region can occur in the absence of antiviral drug selection pressure, which was attributed to mutations in the envelope (S) gene region under immune selective pressure. For instance, I195M in the S protein (sI195M) and sW196S can produce rtM204I and rtL180M/rtM204I in RT, which could confer resistance to LMV (3, 4). Indeed, whether the YMDD motif mutations exist in the treatment-naive patients remains controversial, and divergent opinions exist in some studies regarding whether drug resistance mutations can exist as the natural variation (5–13). Since NA resistance (NA^r^) mutations in the RT region were mostly reported in the posttreatment patients, in this study, we investigated the occurrence frequency of NA^r^ mutations in the absence of the selective pressure of antiviral drugs in Chinese CHB patients by Sanger sequencing and next-generation sequencing (NGS).

Several researchers have analyzed spontaneous mutations by Sanger sequencing, and the results revealed that mutations in pre-S/S, pre-C/C, and X were involved in the progression of liver disease (14–16). Compared with single-site mutation, multiple-site mutations in those genes were complicated and often closely linked to the development of HCC (16, 17). Furthermore, it has been reported that patients with spontaneous YMDD mutations had a higher risk of developing HBV-related HCC, suggesting that spontaneous mutations in the RT region were also related to the progression of liver disease (14). Given that the results were obtained by Sanger sequencing with a limited sensitivity, next-generation sequencing could be considered to investigate further the association between the spontaneous mutations and the progression of liver disease. In addition, the combination of spontaneous mutations in the RT and their clinical significance, especially the association between mutations in RT and the progression of the liver disease, have been relatively less discussed before.

In this study, we analyzed the mutations in the RT region by Sanger sequencing and next-generation sequencing, aiming to characterize the natural amino acid substitutions in the RT region and further analyze their potential clinical significance especially associated with the progression of liver disease.

## MATERIALS AND METHODS

### Patients and blood samples.

A total of 493 treatment-naive HBV-infected patients were enrolled at the First Affiliated Hospital of Fujian Medical University (Fujian, China) from January 2011 to December 2016. Venous blood samples were obtained, and the serum was separated by centrifugation. All serum specimens were stored at −80°C until used. Both Sanger sequencing and next-generation sequencing (NGS) were performed on 63 specimens simultaneously, Sanger sequencing was performed on 427 specimens, and next-generation sequencing was performed on 3 specimens. All patients were diagnosed as having chronic HBV infection according to the criteria suggested by the guideline of prevention and treatment for chronic hepatitis B in 2015 in China (31). Patients coinfected with the hepatitis A/C/D virus or human immunodeficiency virus, or other concomitant liver disease such as primary biliary cirrhosis, autoimmune liver disease, drug abuse, or alcohol abuse were excluded. Written consent was obtained from each patient, and the study was approved by the ethics committee of the First Affiliated Hospital of Fujian Medical University.

### Laboratory tests.

Liver function tests and serum HBV markers were conventionally performed in the clinical laboratory of the First Affiliated Hospital of Fujian Medical University. Serum hepatitis B s antigen (HBsAg), anti-HBs, hepatitis B e antigen (HBeAg), anti-HBe, and anti-HBc were determined on the wholly automatic immune fluorescence analyzer Abbott Type i4000 (Abbott Laboratories, USA) using the original attached commercial kits. Serum alanine aminotransferase (ALT) and aspartate aminotransferase (AST) levels were assayed using an automated biochemical technique (Siemens Healthcare Diagnostics, USA). Serum HBV DNA was quantiﬁed using the TaqMan PCR assay (Sansure Biotech, China) on an ABI 7500 real-time PCR system (Life Technologies, USA) with a lowest detection limit of 20 IU/ml.

### Sanger sequencing.

HBV DNA was extracted from 500-μl serum samples of patients according to the protocol of the TIANamp genomic DNA kit (Tiangen Biotech, Beijing, China). The HBV RT gene was amplified by PCR using forward primer 5′-CTCATGTTGCTGTACAAAACC-3′ (nt 559 to ∼nt 579) and reverse primer 5′-CAATTCKTTGACATACTTTCCA-3′ (nt 1000 to ∼nt 979). The PCR conditions were as follows: 94°C for 5 min; 35 cycles of 94°C for 1 min, 60°C for 1 min, and 72°C for 1 min; and then 72°C for 10 min. The PCR products were purified using a QIAquick gel extraction kit (Qiagen, Germany) and directly sequenced (Beijing Genomics Institute, Shenzhen, China).

### Next-generation sequencing.

The HBV RT gene was amplified by nested PCR using first-round primer pairs (forward, 5′-TAGGACCCCTGCTCGTGTTA-3′; reverse primer, 5′-GCTAGGAGTTCCGCAGTATGG-3′) and second-round primer pairs (forward, 5′-GGGCTTTCCCCCACTGTY-3′; reverse, 5′-GRGCAACGGGGTAAAGGK-3′). Raw data were obtained from the Illumina MiSeq sequencing platform with 2 × 300-bp dual-end mode sequencing, and the average depth was more than 1,500×. Reads with an average quality of >Q20 were able to be used for subsequent analysis. After removing the adapter sequences in the reads, small fragments with a length of less than 25 bp were discarded, with the aim to avoid the contamination of PCR primers. Subsequently, clean data were obtained using the BWA software for the final mutation analysis. The real mutation frequency threshold was 0.889%, according to Yang et al. (18). In brief, any mutation detected from the HBV wild-type (WT) plasmid template by NGS was due to errors. Therefore, after aligning the sequencing read of the HBV WT plasmid template by NGS with that by Sanger sequencing, the mean overall error rate was 0.741% (standard deviation, 0.074%) per base. The frequency threshold was set to two standard deviations higher than the mean error rate, i.e., 0.889%, to differentiate a real mutation from a sequencing error.

### Sequence alignment and HBV genotyping.

Mutations were analyzed by aligning HBV RT sequences with the consensus sequence generated based on the HBV RT sequences in this study and the reference sequences in previous studies according to published literature (6, 19). In brief, all of the sequences in this study were aligned with the previous published HBV wild sequences (20) to obtain the consensus sequence as the reference sequence, and then each of the sequences was aligned with the reference sequence described above to analyze the mutation. Shannon entropy was applied as a measure of variation in RT and calculated by the online tool Entropy (https://www.hiv.lanl.gov/content/sequence/ENTROPY/entropy.html) (21).

HBV genotyping was performed by the phylogenetic analysis using 17 genotype B DNA sequences (accession numbers AY217364, AY217358, AY206387, AY206383, AY206380, AY206375, AY206373, FJ518812, FJ518811, FJ386688, FJ386684, FJ386683, FJ386682, FJ386681, FJ386680, FJ386676, and FJ386675) and 17 genotype C DNA sequences (accession numbers EU916228, AY217372, EU439025, EU439012, EU916232, EU916229, EU916227, EU916226, EU916225, EU916224, EU916223, EU916222, EU916221, EU916220, EU916219, EU916218, and EU916217) in GenBank. An online tool (https://www.ncbi.nlm.nih.gov/projects/genotyping/formpage.cgi) was also applied to verify the accuracy of HBV genotyping.

### Liver inflammation grading and fibrosis staging.

Liver biopsies were performed by clinicians. Then, pathological tissues from the biopsy specimens were analyzed by experienced pathologists. The severity of liver disease was evaluated according to grade of inflammation (G) and stage of fibrosis (S). The results were obtained from the electronic medical records (EMR) system.

### Statistical analysis.

Statistical difference was evaluated by *t* test, Mann-Whitney U test, Kruskal-Wallis test, chi-square test (Fisher’s exact test was used when needed), one-way analysis of variance, or multiple comparisons with IBM SPSS Statistics software (version 22.0.0; IBM, Armonk, NY, USA) where appropriate. All *P* values were two-tailed. A *P* value of <0.05 was considered to be statistically significant.

## RESULTS

### Characterization of mutations in HBV reverse transcriptase gene by Sanger sequencing.

The HBV RT region from rt145 to rt289, covering parts of the A-B interdomain, domain B, B-C interdomain, domain C, C-D interdomain, domain D, D-E interdomain, domain E, and parts of the E-RNA H interdomain, was sequenced by Sanger sequencing in 427 treatment-naive CHB patients, including 57 HBV carriers (HC), 265 chronic hepatitis B (CHB) patients, and 105 patients with advanced liver disease (ALD). Among these 427 patients, the 57 HBV carriers consisted of 39 immunotolerant carriers and 18 inactive carriers, and the 105 ALD patients consisted of 83 LC patients and 22 HCC patients. The characteristics of these patients are shown in Table 1.

**TABLE 1:** Characteristics of 427 patients tested by Sanger sequencing and 66 patients tested by next-generation sequencing

Characteristic	Data for each group by sequence method^*a*^					
	Sanger sequencing (*n* = 427)			Next-generation sequencing (*n* = 66)		
	HC (*n* = 57)	CHB (*n* = 265)	ALD (*n* = 105)	HC (*n* = 15)	CHB (*n* = 41)	ALD (*n* = 10)
Age (mean ± SD) (yr)	35.32 ± 12.80	32.38 ± 11.76	45.10 ± 12.67	40.40 ± 15.38	44.02 ± 13.95	54.60 ± 14.81
No. of males/females	26/31	185/80	88/17	6/9	31/10	9/1
No. HBeAg positive/negative	39/18	188/77	55/50	6/9	13/28	0/10
Serum HBV DNA concn (mean ± SD) (log_10_ IU/ml)	6.50 ± 1.83	6.47 ± 1.38	5.55 ± 1.45	5.37 ± 2.19	5.41 ± 1.39	5.05 ± 1.22
ALT concn (mean ± SD) (U/liter)	28.95 ± 8.89	323.32 ± 407.52	183.08 ± 246.93	24.40 ± 8.00	216.33 ± 258.66	63.9 ± 28.45
No. with genotype B/C	33/24	172/93	41/64	9/6	11/30	4/6

a HC, hepatitis B virus carriers, including immunotolerant carriers and inactive carriers; CHB, chronic hepatitis B; ALD, advanced liver disease, including LC and HCC.

As previously reported (6), 12 genotype-dependent amino acid sites were confirmed in this study (Table 2). The results revealed that the presence of tyrosine or phenylalanine at rt151, tyrosine or phenylalanine at rt221, alanine or threonine at rt222, alanine or serine at rt223, valine or isoleucine at rt224, histidine or asparagine at rt238, glutamine or leucine at rt267, and methionine or glutamine at rt271 was significantly correlated (*P* < 0.0001) with genotype B or C, respectively. Isoleucine at rt191 and histidine at rt226 were significantly more associated with genotype B than genotype C (*P* < 0.0001), respectively, and cysteine at rt256 was significantly more associated with genotype C than genotype B (*P* = 0.003), though valine, asparagine, or serine was predominant at rt191, rt226, or rt256 for both genotypes, respectively. Isoleucine was predominant at rt269 for genotype B (*P* < 0.0001), while isoleucine (36/69) and leucine (33/69) were comparable at rt269 for genotype C. In addition, although methionine was predominant at rt271 for genotype B, leucine at rt271 was significantly more associated with genotype B than with genotype C (*P* < 0.001). According to previous investigations (6, 19), the consensus amino acid residue at each of above-described sites was genotype dependent and regarded as the reference. The other residues at these sites present at a low frequency were regarded as spontaneous mutations in this study.

**TABLE 2:** Genotype-dependent amino acid polymorphic sites found in this study

RT	Mutation^*a*^	No. of polymorphic sites in genotype:		*P* value
		B	C	
rt151	Y	62	1	<0.0001
	F	0	39	
rt191	V	245	167	<0.0001
	I	1	13	
rt221	Y	244	12	<0.0001
	F	1	174	
	C^*b*^	1	0	
	H^*b*^	2	0	
rt222	A	177	4	<0.0001
	T	71	182	
rt223	A	246	32	<0.0001
	S	2	153	
	T^*b*^	0	1	
rt224	V	241	27	<0.0001
	I	2	156	
	L^*b*^	4	0	
	T^*b*^	0	2	
rt226	N	225	177	<0.0001
	H	19	0	
	T^*b*^	2	3	
rt238	H	242	14	<0.0001
	N	2	168	
	Q^*b*^	2	1	
	A^*b*^	0	1	
	S^*b*^	0	2	
rt256	S	194	100	0.003
	C	7	14	
	G^*b*^	8	0	
rt267	Q	95	9	<0.0001
	L	4	60	
	H^*b*^	3	0	
rt269	I	99	36	<0.0001
	L	1	33	
rt271	M	78	1	<0.0001
	Q	4	65	
	L	15	0	
	K^*b*^	1	0	
	E^*b*^	0	1	
	H^*b*^	0	1	
	V^*b*^	1	0	
	I^*b*^	1	0	

a Y, tyrosine; F, phenylalanine; C, cysteine; H, histidine; A, alanine; T, threonine; S, serine; V, valine; I, isoleucine; L, leucine; N, asparagine; Q, glutamine; G, glycine; M, methionine; K, lysine; E, glutamic acid.

b Described as naturally occurring polymorphic mutations in this study.

On the basis of the consensus amino acid residue mentioned above, mutations in this study were classified into 4 categories: primary resistance mutations, secondary resistance mutations, putative resistance mutations, and pretreatment mutations, according to previous studies (6, 22). Primary and secondary resistance mutations are well known as classical antiviral resistance mutations which have been confirmed by phenotypic experiments *in vitro* and vivo, while putative resistance mutations and pretreatment mutations remained to be proven by experiments. Moreover, putative resistance mutations were related to long-term NA therapy, and pretreatment mutations were mainly found in treatment-naive patients. The results in this study showed that mutations in the RT were found in 36.53% (156/427) of the isolates, and mutations at 56 amino acid residue sites were involved, including 4 in the A-B interdomain, 8 in domain B, 1 in the B-C interdomain, 3 in domain C, 15 in the C-D interdomain, 5 in domain D, 3 in the D-E interdomain, 11 in domain E, and 6 in the E-RNA H interdomain (Table 3). Of these 56 amino acid residue sites, mutations in 22 sites within the overlapping region of RT and S led to amino acid mutations in the S region at the same time. Notably, mutations in 36 amino acid residue sites (36/56 [64.29%]) could change B or T cell epitopes (23, 24) in the RT or S protein, and these 36 sites included 4 in the A-B interdomain (4/4 [100%]), 7 in domain B (7/8 [87.5%]), 1 in the B-C interdomain (1/1 [100%]), 3 in domain C (3/3 [100%]), 12 in the C-D interdomain (12/15 [80%]), 3 in domain D (3/5 [60%]), 0 in the D-E interdomain (0/3 [0%]), 0 in domain E (0/11 [0%]), and 6 in the E-RNA H interdomain (6/6 [100%]). In addition, except for only 1 patient with a mutation at rt181 (A181T) in the ALD group, there was not any primary resistance mutation (i.e., I169T, T184A/C/F/G/I/L/M/S, A194T, S202C/G/I, M204I/V/S, N236T, or M250I/L/V) and secondary resistance mutation (i.e., V173L or L180M) found in treatment-naive patients, while 9 putative resistance mutations and 51 pretreatment mutations were detected in these patients.

**TABLE 3:** Mutations of HBV reverse transcriptase analyzed in this study

RT mutation by type	RT region	No. of mutations/no. of amino acids			Epitope			Effect on HBsAg	No. of mutations/no. of amino acids			Epitope		
		HC	CHB	ALD	B cell epitope	T cell epitope			HC	CHB	ALD	B cell epitope	T cell epitope	
						HLA I	HLA II						HLA I	HLA II
Primary resistance mutations^*a*^														
rtA181T	B	0	0	1/105				sW172stop	0	0	1/105	√	√	√
Putative resistance mutations^*b*^														
rtV207I	C	0	1/265	0		√		sM198I/sW199stop	0	1/265	0	√		
rtS213T	C-D	1/57	14/265	6/105				sS204R	1/57	14/265	6/105	√		
rtV214A	C-D	1/57	2/265	3/105				sY206H	1/57	2/265	3/105	√		
rtE218D	C-D	0	1/265	0				sS210M	0	1/265	0		√	
rtL229V	D	0	5/265	1/105		√		sF220L	0	5/265	1/105			√
rtI233V	D	0	0	1/105		√		—^*e*^	0	0	0			
rtN238S^*c*^	D	0	2/265	0				—^*f*^	—^*f*^	—^*f*^	—^*f*^			
rtY245H	D-E	0	1/264	0				—^*f*^	—^*f*^	—^*f*^	—^*f*^			
rtS256G^*c*^	E	3/57	4/254	1/103				—^*f*^	—^*f*^	—^*f*^	—^*f*^			
Pretreatment mutations^*d*^														
rtR153Q	A-B	0	1/42	0				sG145R	0	1/42	0	√		√
rtV207M	C	3/57	2/265	1/105		√		sM198I	3/57	2/265	1/105	√		
rtH238Q^*c*^	D	0	1/265	1/105				—^*f*^	—^*f*^	—^*f*^	—^*f*^			
rtK154Q	A-B	0	1/42	0			√	—^*e*^	0	0	0			
rtL157M	A-B	0	1/77	0			√	—^*e*^	0	0	0			
rtI163V	A-B	2/49	1/94	0			√	—^*e*^	0	0	0			
rtL164M/F	B	1/49	1/94	2/31			√	sW156L	0	1/94	1/31		√	
rtV173M	B	1/56	3/241	0				—^*e*^	0	0	0			
rtP177L	B	0	1/265	0				sR169C	0	1/265	0	√		√
rtF178C	B	0	1/265	0				sF170V	0	1/265	0	√		√
rtS185R	B	0	0/265	3/105		√		—^*e*^	0	0	0			
rtI187V/L	B	1/57	3/265	1/105		√		—^*e*^	0	0	0			
rtV190L	B	0	1/265	0		√		sQ181H	0	1/265	0	√		
rtC198W	B-C	0	1/265	0				sV190G	0	1/265	0	√	√	
rtL199V	C	1/57	1/265	4/105				—^*e*^	0	0	0			
rtV207L	C	0	1/265	1/105		√		sM198I	0	1/265	1/105	√		
rtL209V	C	1/59	0	0		√		sF200L	1/59	0	0	√		
rtK212N/T	C-D	1/57	1/265	0				sS204T/R	1/57	1/265	0	√		
rtQ215H	C-D	0	0	1/105				sN207T	0	0	1/105	√	√	
rtL217H	C-D	0	0	1/105				sL209M	0	0	1/105		√	
rtS219A	C-D	1/57	8/265	3/105				sS210R/K	1/57	8/265	3/105		√	
rtL220I/V	C-D	2/57	7/265	6/105				—^*e*^	0	0	0			
rtY221H/C^*c*^	C-D	0	3/265	0				sM213V	0	1/265	0		√	
rtS223T^*c*^	C-D	0	1/265	0				—^*e*^	0	0	0			
rtV/I224L/T^*c*^	C-D	0	6/265	0				—^*e*^	0	0	0			
rtT225S/I	C-D	0	3/265	1/105				sL216F/sP217L	0	3/265	1/105		√	√
rtN226T^*c*^	C-D	1/57	3/265	1/105		√		sI218L	1/57	3/265	1/105			√
rtF227Y	C-D	0	0	1/105		√		sF219I	0	0	1/105			√
rtL228I/F/R	C-D	0	2/265	0		√		sF219L/sF220V	0	2/265	0			√
rtL229M	D	1/57	1/265	0		√		sF220L	1/57	1/265	0			√
rtG232D	D	0	0	1/105		√		sV224I	0	0	1/105			
rtN238Q/A^*c*^	D	0	1/265	1/105				—^*f*^	—^*f*^	—^*f*^	—^*f*^			
rtK241N	D	0	1/265	0				—^*f*^	—^*f*^	—^*f*^	—^*f*^			
rtR242S	D-E	0	0	1/105				—^*f*^	—^*f*^	—^*f*^	—^*f*^			
rtW243G	D-E	0	1/264	0				—^*f*^	—^*f*^	—^*f*^	—^*f*^			
rtS246P	E	0	0	1/104				—^*f*^	—^*f*^	—^*f*^	—^*f*^			
rtN248H	E	1/57	8/263	2/104				—^*f*^	—^*f*^	—^*f*^	—^*f*^			
rtF249I	E	0	1/263	1/104				—^*f*^	—^*f*^	—^*f*^	—^*f*^			
rtM250R	E	0	2/263	0				—^*f*^	—^*f*^	—^*f*^	—^*f*^			
rtV253I	E	2/57	4/261	0				—^*f*^	—^*f*^	—^*f*^	—^*f*^			
rtI254S/M	E	0	2/260	0				—^*f*^	—^*f*^	—^*f*^	—^*f*^			
rtT259S	E	0	1/90	0				—^*f*^	—^*f*^	—^*f*^	—^*f*^			
rtE263D	E	2/50	1/89	1/30				—^*f*^	—^*f*^	—^*f*^	—^*f*^			
rtV266L/I/E	E	1/50	4/89	2/30				—^*f*^	—^*f*^	—^*f*^	—^*f*^			
rtQ267H^*c*^	E	0	3/89	0				—^*f*^	—^*f*^	—^*f*^	—^*f*^			
rtM/Q271K/I/E/H/V^*c*^	E-RNA H	1/50	3/87	2/29		√		—^*f*^	—^*f*^	—^*f*^	—^*f*^			
rtR274K/Q	E-RNA H	3/50	2/79	3/26		√		—^*f*^	—^*f*^	—^*f*^	—^*f*^			
rtK275Q	E-RNA H	2/50	0	0		√		—^*f*^	—^*f*^	—^*f*^	—^*f*^			
rtL276H/F	E-RNA H	0	1/51	3/21		√		—^*f*^	—^*f*^	—^*f*^	—^*f*^			
rtV278I	E-RNA H	1/49	0	0		√		—^*f*^	—^*f*^	—^*f*^	—^*f*^			
rtI282V	E-RNA H	1/21	1/48	0				—^*f*^	—^*f*^	—^*f*^	—^*f*^			

a Well-known NA resistance mutations (primary and secondary) with phenotypic data. No secondary resistance mutations were found.

b Putative mutations that were relevant to NA resistance but not experimentally confirmed.

c Genotype-dependent amino acid polymorphic positions identified in this study.

d Pretreatment mutations that were found in NA-naive patients but not experimentally confirmed.

e Not determined.

f Not applicable.

### Mutation distribution and frequency in different RT sections.

**FIG 1:**
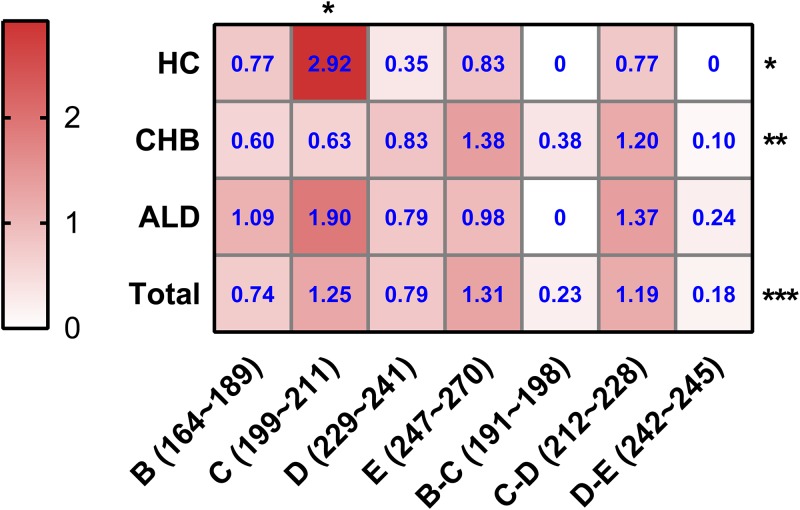
Heat map for mutation frequencies of different sections of the HBV RT region. HC, HBV carriers; CHB, chronic hepatitis B; ALD, advanced liver disease. The mutation frequency for each domain is calculated by the number of mutations in each domain/total number of sites in that domain; values in blue font in each cell represent 100× mutation frequency of that section in the HC, CHB, or ALD group; *, *P* < 0.05; **, *P* < 0.01; ***, *P* < 0.001.

To testify the difference of the mutation distribution among 3 groups (HC group, CHB group, and ALD group), the mutation frequency for each section of the RT region was calculated, referring to the previous study (6). For instance, the mutation frequency of domain D in the CHB group was calculated with the following formula: 11 mutations detected/(5 studied sites × 265 isolates) × 100% = 11/1,325 × 100% = 0.83%. The detailed results for each section of the RT region in different groups are shown in Fig. 1 . In total, the mutations analyzed in this study occurred mainly in domain C, the C-D interdomain, and domain E. In the HC group, the mutation frequency of domain C (2.92%) was highest (*P* < 0.05). In the CHB group, significantly higher mutation frequencies were found in domain E (1.38%) and the C-D interdomain (1.20%) than in other sections (*P* < 0.01). In the ALD group, domain C showed the higher mutation frequency (1.90%), though this difference was not statistically significant. Notably, among these 3 groups, the mutation frequency of domain C was highest in the HC group, while it was lowest in the CHB group (*P* < 0.05).

### Correlation between HBV RT mutations and clinical features.

To investigate the clinical influence of HBV mutations in treatment-naive patients, the clinical characteristics of patients were compared between 271 patients without mutations and 156 patients comprising 115 patients with a single mutation and 41 patients with multiple mutations. The results showed that patients with mutations, especially with multiple mutations, were significantly older and had significantly lower HBeAg-positive ratio, HBV DNA loads, and levels of HBsAg quantification than those without mutations (Table 4).

**TABLE 4:** Comparison of patients with and without HBV RT mutations

Characteristic	Nonmutation (*n* = 271)	Mutation			*P*^*a*^
		Single mutation (*n* = 115)	Multimutation (*n* = 41)	Total (*n* = 156)	
Age (mean ± SD) (yr)	34.71 ± 12.99	37.20 ± 13.86	40.12 ± 12.55^*b*^	37.97 ± 13.55	0.014
No. of males/females	187/84	81/34	31/10	112/44	0.544
No. HBeAg positive/negative	191/80	74/41	15/26^*b*^^,^^*c*^	89/67	0.005
Serum HBV DNA concn (mean ± SD) (log_10_ IU/ml)	6.36 ± 1.45	6.20 ± 1.60	5.57 ± 1.70^*b*^	6.05 ± 1.64	0.057
HBsAg concn (mean ± SD) (log_10_ IU/ml)	3.63 ± 0.83	2.73 ± 1.48^*d*^	2.05 ± 1.70^*b*^^,^^*c*^	2.55 ± 1.56	0.000
Anti-HBcAg(mean ± SD) (S/CO)	12.46 ± 3.77	11.80 ± 2.66	11.87 ± 2.76	11.82 ± 2.67	0.100
ALT concn (mean ± SD) (U/liter)	252.46 ± 373.41	248.92 ± 340.58	226.21 ± 332.09	243.01 ± 337.43	0.800
AST concn (mean ± SD) (U/liter)	152.89 ± 312.97	144.96 ± 205.48	102.83 ± 144.75	134.07 ± 192.08	0.505
No. of genotype B/C	159/112	61/54	26/15	87/69	0.559

a Nonmutation versus mutation.

b *P* < 0.0005, single mutation versus nonmutation.

c *P* < 0.05, multimutation versus nonmutation.

d *P* < 0.05, multimutation versus single mutation.

**FIG 2:**
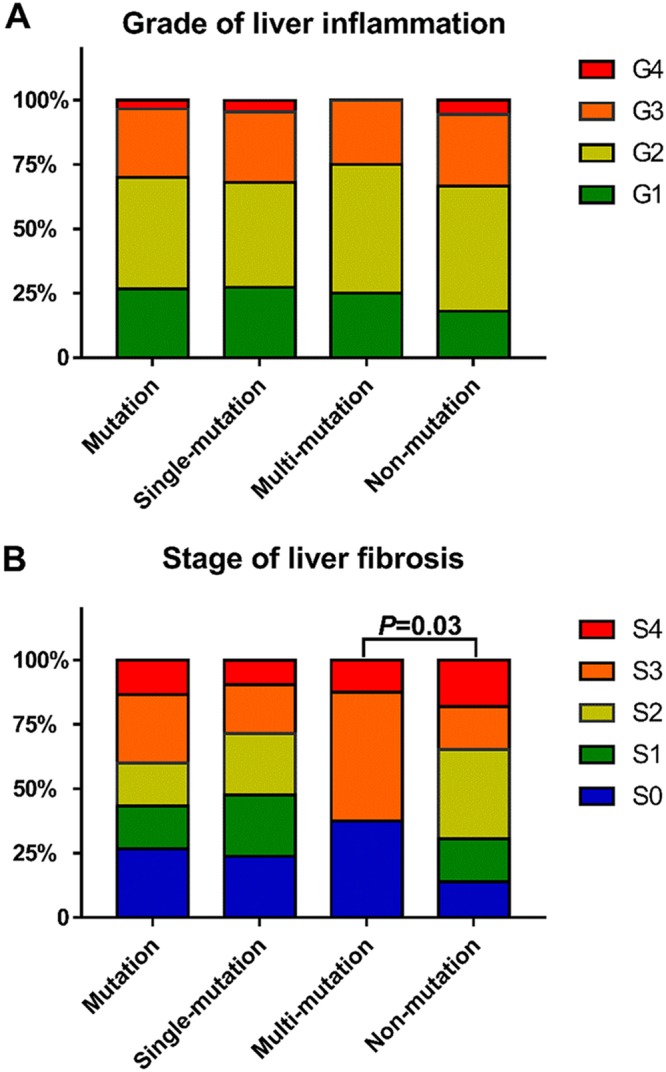
(A) Pattern of distribution according to grade of liver inflammation based on the number of HBV RT mutations. G1, portal area inflammation; G2, mild piecemeal necrosis (PN); G3, moderate PN; G4, severe PN. (B) Pattern of distribution according to stage of liver fibrosis based on the number of HBV RT mutations. S0, no fibrosis; S1, fibrosis was limited to the area around the liver sinuses and in the hepatic lobule; S2, fibrous septum was formed, but hepatic lobule structures remained; S3, fibrous septum was formed and hepatic lobules were disorganized; S4, liver cirrhosis.

Additionally, to investigate the relationship between the severity of liver disease and RT mutations, the results of liver biopsies from 102 patients including 72 patients without RT mutations, 22 patients with a single RT mutation, and 8 patients with multiple RT mutations were analyzed. The severity of liver disease was evaluated according to the grade of inflammation (G) and the stage of fibrosis (S) (Fig. 2). The results showed that there was no significantly difference in the grade of inflammation among nonmutation group, single-mutation group, and multimutation group. Although no significant difference was found between the nonmutation group and single-mutation group, a significant difference in the stage of fibrosis was found between the nonmutation group and multimutation group (*P* = 0.030), indicating that patients with multiple mutations in the RT region had a greater risk of developing more serious liver fibrosis.

### Comparison of mutant characterization in HBV reverse transcriptase gene by next-generation sequencing and Sanger sequencing.

**FIG 3:**
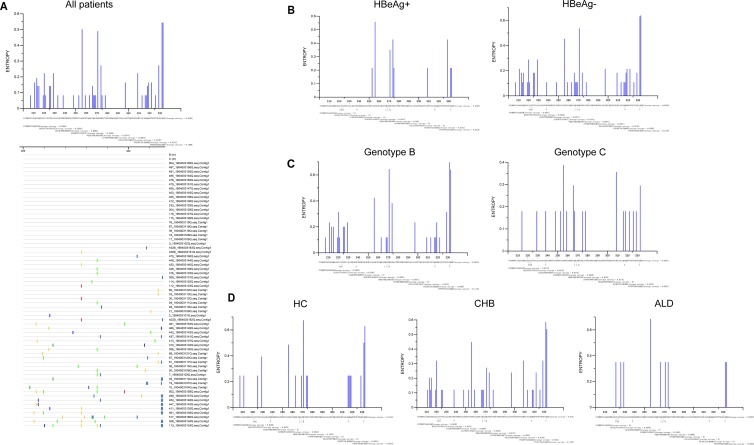
Characteristics of mutations tested by Sanger sequencing in RT region represented by Shannon entropy. (A) Characteristics of mutations in 63 patients who were tested by NGS at the same time. (B to D) Distribution of mutations in patients with different HBeAg statuses, HBV genotype infections, and liver disease stages.

Due to the limited sensitivity of Sanger sequencing, next-generation sequencing was performed on 66 treatment-naive patients with chronic HBV infection from rt201 to rt335 (Table 1), and Sanger sequencing was also performed on 63 of these 66 patients for comparison (Fig. 3).

**FIG 4:**
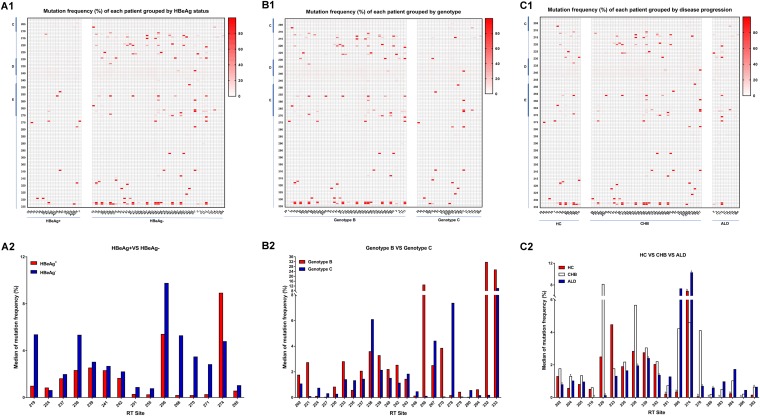
Mutation frequency of each patient and median of mutation frequency at particular RT sites by next-generation sequencing. HC, HBV carriers; CHB, chronic hepatitis B; ALD, advanced liver disease. (A1 to C1) Heat map of mutation frequency in patients grouped by HBeAg status, genotype, or disease progression (*n* = 19 for HBeAg-positive patients and *n* = 47 for HBeAg-negative patients; *n* = 43 for patients infected by genotype B HBV and *n* = 23 for patients infected by genotype C HBV; *n* = 15 for chronic HC, *n* = 41 for patients with CHB, and *n* = 10 for patients with ALD). (A2) Significantly different distributions at particular RT sites between HBeAg-positive patients and HBeAg-negative patients (for A2 to C2, the *x* axis represents RT sites at which mutation frequency was significantly different and the *y* axis represents the median of mutation frequency). (B2) Significantly different distributions at particular RT sites between patients infected by genotype B or genotype C HBV. (C2) Significantly different distributions at particular RT sites among HC, CHB, and ALD group (a, *P* < 0.05, HC group versus CHB group; b, *P* < 0.05, HC group versus ALD group; c, *P* < 0.05, CHB group versus ALD group).

The results tested by NGS were consistent with those by the conventional Sanger sequencing (Fig. 3 and 4). It showed that mutations mainly clustered in rt212 to ∼rt228 located in the C-D interdomain, rt247 to ∼rt270 located in domain E, and rt295 to ∼rt335 located in the E-RNA H interdomain (Fig. 3A and 4A1 to C1). Moreover, the mutations seemingly tended to occur in patients with negative HBeAg, genotype B HBV, or CHB (Fig. 3B to D and 4A1 to C1).

Considering that the low sensitivity of Sanger sequencing limited the detection of NA^r^ mutations with a low rate (<20%), 4 classical drug resistance mutations (S202C/G/I, M204I/V/S, N236T, and M250I/L/V) and 12 putative NA^r^ mutations (V207I, S213T, V214A, Q215P/S, L217R, E218D, L229G/V/W/F, I233V, P237H, N/H238D/S/T, Y245H, and S/C256G) tested by NGS and Sanger sequencing were compared (Table 5). The classical drug resistance mutations were not found by Sanger sequencing, while S202C/G/I (0.92% to ∼3.45%), M204I/V/S (0.90% to ∼8.32%), N236T (1.16% to ∼3.63%), and M250I/L/V (0.90% to ∼1.83%) were found at a low rate by NGS. Also, rtS213T, rtV214A, rtL229G/V/W/F, N/H238D/T, and S/C256G were detected in 13 patients by both Sanger sequencing and NGS, including 3 patients with rtS213T (98.44%, 46.88%, and 80.97% by NGS), 2 with rtV214A (49.18% and 99.24%), 1 with rtL229V (50.84%), 1 with rtN238T (98.85%), 5 with S/C256G (97.28%, 97.93%, 92.26%, 97.79%, and 98.31%), and 1 with rtL229V plus rtS/C256G (82.36% for rtL229V and 74.81% for rtS/C256G). Notably, 3 putative NA^r^ mutations (rtL229V, H238Q, and S/C256G) with rates between 20% and 25% tested by NGS in patients 3, 20, and 497, respectively, were not detected by Sanger sequecing, implying the limitation of Sanger sequencing further.

**TABLE 5:** Mutations associated with antiviral therapy analyzed by Sanger sequencing and next-generation sequencing

Patient no.	Sequencing method	Classical drug resistance mutations				Putative NA^r^ mutations											
		S202C/G/I	M204I/V/S	N236T	M250I/L/V	V207I	S213T	V214A	Q215P/S	L217R	E218D	L229G/V/W/F	I233V	P237H	N/H238D/S/T	Y245H	S/C256G
2	Sanger	−	−	−	−	−	−	−	−	−	−	−	−	−	−	−	−
	NGS (%)	1.00	0.00	1.88	1.83	0.00	12.47	0.00	1.18	0.00	0.98	0.96	1.45	1.57	2.98	9.50	0.00
3	Sanger	−	−	−	−	−	−	−	−	−	−	−	−	−	−	−	−
	NGS (%)	1.41	0.00	1.94	0.00	0.00	0.00	0.00	1.20	0.00	0.00	21.37	1.40	1.40	2.61	1.65	0.00
7	Sanger	−	−	−	−	−	−	−	−	−	−	−	−	−	−	−	−
	NGS (%)	1.21	1.18	1.33	0.00	0.00	0.00	0.00	1.57	0.00	1.20	0.00	1.38	1.54	3.02	1.69	0.00
10	Sanger	−	−	−	−	−	−	−	−	−	−	−	−	−	−	−	−
	NGS (%)	1.18	0.90	1.54	0.00	0.00	0.00	0.00	1.08	0.00	1.14	0.00	1.10	1.14	2.17	1.71	0.00
17	Sanger	−	−	−	−	−	−	−	−	−	−	−	−	−	−	−	−
	NGS (%)	0.00	0.00	1.34	0.00	0.00	0.00	0.00	1.13	0.00	0.92	0.00	0.00	1.01	1.48	1.31	0.00
18	Sanger	−	−	−	−	−	−	−	−	−	−	−	−	−	−	−	−
	NGS (%)	1.60	1.40	1.56	0.00	0.00	0.00	0.00	1.70	0.00	1.01	0.00	1.56	1.18	2.60	1.93	1.48
19	Sanger	−	−	−	−	−	−	−	−	−	−	−	−	−	−	−	−
	NGS (%)	1.05	0.00	1.62	0.00	0.00	0.00	0.00	1.15	0.00	0.00	0.00	0.99	1.36	3.00	1.82	3.93
20	Sanger	−	−	−	−	−	−	−	−	−	−	−	−	−	−	−	−
	NGS (%)	1.54	0.95	1.87	0.00	0.00	0.00	0.00	1.03	0.00	1.11	0.00	1.26	1.30	2.43	1.71	23.49
21	Sanger	−	−	−	−	−	−	−	−	−	−	−	−	−	−	−	−
	NGS (%)	1.73	1.11	1.73	0.00	1.26	3.71	0.00	1.17	0.00	0.95	0.00	1.62	1.68	2.21	1.85	0.00
28	Sanger	−	−	−	−	−	−	−	−	−	−	−	−	−	−	−	−
	NGS (%)	0.00	0.00	1.37	0.00	0.00	0.00	0.00	1.43	0.00	0.00	0.00	1.21	1.39	1.46	1.95	0.00
29	Sanger	−	−	−	−	−	−	−	−	−	−	−	−	−	−	−	−
	NGS (%)	1.28	0.00	1.55	0.95	0.00	0.00	0.00	1.57	0.00	1.05	0.00	1.55	1.16	2.98	1.49	0.00
30	Sanger	−	−	−	−	−	−	−	−	−	−	−	−	−	−	−	−
	NGS (%)	1.06	0.92	1.63	0.00	0.00	0.00	0.00	1.34	0.00	0.00	0.00	1.52	1.00	1.89	2.23	0.00
33	Sanger	−	−	−	−	−	−	−	−	−	−	−	−	−	−	−	−
	NGS (%)	0.00	0.00	1.52	0.00	0.00	0.00	0.00	0.00	0.00	0.95	0.00	1.15	1.21	1.28	1.96	0.00
35	Sanger	−	−	−	−	−	−	−	−	−	−	+	−	−	−	−	−
	NGS (%)	1.18	0.00	1.53	0.00	0.00	0.00	0.00	1.18	0.00	0.00	50.84	0.93	1.18	2.17	1.30	0.00
36	Sanger	−	−	−	−	−	−	−	−	−	−	−	−	−	−	−	−
	NGS (%)	0.00	0.00	1.39	0.00	0.00	0.00	0.00	0.00	0.00	0.00	0.00	1.72	0.00	1.64	1.00	0.00
39	Sanger	−	−	−	−	−	−	−	−	−	−	−	−	−	−	−	−
	NGS (%)	0.00	0.00	1.34	0.00	0.00	0.00	0.00	0.94	0.00	0.00	0.00	1.22	0.92	1.57	1.34	0.00
61	Sanger	−	−	−	−	−	−	−	−	−	−	−	−	−	−	−	−
	NGS (%)	1.44	2.01	1.70	0.98	0.00	0.00	0.00	1.05	0.00	1.16	0.00	1.26	1.37	2.16	1.68	0.00
67	Sanger	−	−	−	−	−	−	−	−	−	−	−	−	−	−	−	−
	NGS (%)	1.22	1.19	1.41	0.00	0.00	0.00	0.00	1.34	0.00	1.15	0.00	1.29	0.00	1.44	1.34	0.00
76	Sanger	−	−	−	−	−	−	−	−	−	−	−	−	−	−	−	−
	NGS (%)	0.00	0.00	1.62	0.00	0.00	0.00	0.00	1.15	0.00	0.00	0.00	1.00	1.00	1.41	1.68	0.00
87	Sanger	−	−	−	−	−	−	+	−	−	−	−	−	−	−	−	−
	NGS (%)	1.37	0.00	1.76	0.00	1.02	0.00	49.18	1.17	0.00	3.42	0.00	1.29	1.35	2.66	1.41	0.98
88	Sanger	−	−	−	−	−	−	−	−	−	−	−	−	−	−	−	−
	NGS (%)	0.92	0.90	1.46	0.00	0.00	0.00	2.43	3.33	0.00	1.00	1.02	1.72	1.21	2.45	2.09	0.00
89	Sanger	−	−	−	−	−	−	−	−	−	−	−	−	−	−	−	−
	NGS (%)	1.15	0.00	1.77	0.00	0.00	0.00	0.00	1.39	0.00	0.00	0.00	1.58	1.15	2.40	1.73	0.00
112	Sanger	−	−	−	−	−	−	−	−	−	−	−	−	−	−	−	−
	NGS (%)	1.19	1.68	1.39	1.06	0.00	0.00	0.00	1.28	0.00	1.33	0.00	1.24	1.02	1.66	1.99	0.00
113	Sanger	−	−	−	−	−	−	−	−	−	−	+	−	−	−	−	+
	NGS (%)	1.22	1.07	1.89	0.00	0.00	0.00	0.00	2.70	0.00	3.88	82.36	1.99	1.58	2.24	1.89	74.81
114	Sanger	−	−	−	−	−	−	−	−	−	−	−	−	−	−	−	−
	NGS (%)	0.00	1.36	1.16	1.07	29.87	0.00	5.75	0.00	0.00	0.00	0.00	1.02	1.09	1.50	1.53	0.00
115	Sanger	−	−	−	−	−	−	−	−	−	−	−	−	−	−	−	−
	NGS (%)	0.97	1.04	1.86	0.00	0.00	0.00	12.96	1.32	0.00	1.23	0.00	1.38	1.35	1.51	1.54	0.00
116	Sanger	−	−	−	−	−	−	−	−	−	−	−	−	−	−	−	−
	NGS (%)	0.00	1.38	1.56	1.11	0.00	0.00	0.00	1.11	0.00	1.11	0.00	1.68	1.47	1.68	2.10	0.00
204	Sanger	−	−	−	−	−	−	−	−	−	−	−	−	−	−	−	−
	NGS (%)	2.26	0.00	2.11	0.00	0.00	1.79	0.00	1.25	1.02	1.51	1.04	1.85	2.70	4.50	2.05	0.00
233	Sanger	−	−	−	−	−	−	−	−	−	−	−	−	−	−	−	−
	NGS (%)	2.21	0.99	2.64	0.90	0.00	0.00	0.00	1.55	0.00	1.53	1.35	1.69	1.92	1.48	1.85	0.00
308	Sanger	−	−	−	−	−	−	−	−	−	−	−	−	−	−	−	−
	NGS (%)	2.33	1.01	2.48	0.00	0.00	0.00	0.00	1.83	1.02	1.46	1.01	2.09	2.48	3.95	2.07	0.00
309	Sanger	−	−	−	−	−	−	−	−	−	−	−	−	−	−	−	−
	NGS (%)	1.46	1.15	1.95	0.00	0.00	0.00	1.46	2.21	1.07	1.33	2.26	1.86	2.17	5.63	1.25	0.00
310	Sanger	−	−	−	−	−	−	−	−	−	−	−	−	−	−	−	−
	NGS (%)	2.21	0.00	2.39	0.94	0.00	0.00	9.13	2.95	1.40	1.24	0.00	1.50	2.31	3.18	1.75	0.00
316	Sanger	−	−	−	−	−	−	−	−	−	−	−	−	−	−	−	−
	NGS (%)	2.12	0.96	2.74	0.92	0.00	1.82	19.72	1.90	0.92	1.85	5.56	4.19	2.62	3.60	1.85	3.58
326	Sanger	−	−	−	−	−	−	−	−	−	−	−	+	−	−	−	+
	NGS (%)	2.69	1.38	3.50	1.23	0.00	1.14	0.00	1.88	1.50	1.90	1.05	43.28	3.88	4.22	2.82	97.28
411	Sanger	−	−	−	−	−	−	−	−	−	−	−	−	−	−	−	+
	NGS (%)	2.26	1.39	2.85	0.00	1.03	0.00	0.00	2.03	0.00	1.98	1.30	1.91	2.51	3.51	2.46	97.93
412	Sanger	−	−	−	−	−	−	−	−	−	−	−	−	−	−	−	−
	NGS (%)	2.48	1.23	2.22	0.00	1.00	1.12	0.00	1.80	1.16	1.67	0.94	1.77	2.92	4.19	2.20	0.00
413	Sanger	−	−	−	−	−	−	+	−	−	−	−	−	−	−	−	−
	NGS (%)	3.36	2.48	2.76	1.49	0.00	0.00	99.24	1.57	0.00	1.91	1.60	1.49	1.91	2.08	3.06	0.00
425	Sanger	−	−	−	−	−	−	−	−	−	−	−	−	−	−	−	−
	NGS (%)	3.45	0.93	2.57	0.00	1.15	0.00	0.00	1.59	0.98	1.57	1.05	1.57	2.53	4.88	2.22	0.98
426	Sanger	−	−	−	−	−	−	−	−	−	−	−	−	−	−	−	−
	NGS (%)	1.38	0.00	2.50	0.00	0.00	0.00	0.00	1.23	1.13	1.37	0.93	1.60	2.07	3.61	1.74	0.00
433	Sanger	−	−	−	−	−	−	−	−	−	−	−	−	−	−	−	+
	NGS (%)	1.38	0.00	2.50	0.00	0.00	0.00	0.00	1.23	1.13	1.37	0.93	1.60	2.07	3.61	1.74	92.26
437	Sanger	−	−	−	−	−	−	−	−	−	−	−	−	−	−	−	−
	NGS (%)	2.74	1.58	3.46	1.05	0.00	1.08	0.00	2.48	1.28	2.13	1.71	3.01	3.66	4.02	3.31	3.01
442	Sanger	−	−	−	−	−	−	−	−	−	−	−	−	−	−	−	−
	NGS (%)	2.00	2.58	3.20	1.73	0.00	0.94	0.00	1.64	0.96	1.75	1.21	1.41	2.62	2.11	3.45	1.24
447	Sanger	−	−	−	−	−	+	−	−	−	−	−	−	−	−	−	−
	NGS (%)	3.35	1.40	3.31	0.00	0.00	98.44	0.00	0.00	0.94	1.75	1.10	5.04	3.80	4.29	2.79	2.08
448	Sanger	−	−	−	−	−	−	−	−	−	−	−	−	−	−	−	−
	NGS (%)	3.24	1.42	3.20	0.00	0.00	0.95	0.00	2.07	1.42	2.26	0.00	2.00	4.29	4.33	2.95	3.24
453	Sanger	−	−	−	−	−	−	−	−	−	−	−	−	−	−	−	−
	NGS (%)	3.38	2.41	3.63	1.83	1.27	1.15	0.00	2.80	1.39	2.04	1.22	2.09	4.06	4.72	2.92	2.78
454	Sanger	−	−	−	−	−	−	−	−	−	−	−	−	−	−	−	−
	NGS (%)	2.65	3.14	2.87	1.61	0.00	0.00	0.00	0.00	0.00	2.35	0.00	1.99	3.25	4.35	3.14	2.47
455	Sanger	−	−	−	−	−	−	−	−	−	−	−	−	−	−	−	−
	NGS (%)	2.57	2.12	3.01	1.03	0.00	0.00	0.00	2.05	1.03	1.44	1.40	2.16	2.57	1.85	2.67	1.10
461	Sanger	−	−	−	−	−	−	−	−	−	−	−	−	−	−	−	+
	NGS (%)	1.59	1.16	1.85	1.14	1.12	0.00	15.73	2.95	0.00	1.18	0.00	2.04	1.74	2.80	10.19	97.79
466	Sanger	−	−	−	−	−	−	−	−	−	−	−	−	−	−	−	−
	NGS (%)	1.47	1.70	1.72	0.00	0.93	0.00	0.00	1.19	0.00	0.00	0.00	1.21	1.61	1.93	2.03	0.00
470	Sanger	−	−	−	−	−	−	−	−	−	−	−	−	−	−	−	−
	NGS (%)	1.18	1.82	1.34	0.00	0.00	0.00	0.00	1.42	0.00	0.00	0.00	1.26	1.12	3.29	2.09	0.00
475	Sanger	−	−	−	−	−	−	−	−	−	−	−	−	−	−	−	−
	NGS (%)	1.61	0.00	1.78	0.00	0.00	0.00	0.00	1.09	0.00	1.35	0.00	1.81	1.65	3.22	2.04	0.00
478	Sanger	−	−	−	−	−	−	−	−	−	−	−	−	−	−	−	−
	NGS (%)	0.94	2.00	1.59	1.46	0.00	0.00	0.00	1.01	0.00	0.00	1.07	1.35	1.07	1.56	1.69	0.00
481	Sanger	−	−	−	−	−	+	−	−	−	−	−	−	−	−	−	−
	NGS (%)	1.51	1.65	2.11	1.51	1.04	46.88	0.00	1.48	0.00	1.13	0.00	2.17	1.92	2.94	1.59	1.07
485	Sanger	−	−	−	−	−	−	−	−	−	−	−	−	−	−	−	−
	NGS (%)	1.39	1.04	1.72	0.00	0.00	0.00	0.00	1.06	0.00	0.00	0.00	1.34	1.29	2.15	2.17	0.00
491	Sanger	−	−	−	−	−	−	−	−	−	−	−	−	−	−	−	−
	NGS (%)	1.83	2.05	2.05	0.00	1.08	0.00	0.89	1.71	0.00	1.83	0.00	1.94	2.16	3.28	2.09	1.16
497	Sanger	−	−	−	−	−	−	−	−	−	−	−	−	−	−	−	−
	NGS (%)	1.58	0.00	1.80	0.00	0.96	0.92	0.00	1.36	1.01	1.23	1.01	1.75	2.54	22.27	2.02	1.18
499	Sanger	−	−	−	−	−	−	−	−	−	−	−	−	−	−	−	−
	NGS (%)	1.11	0.00	1.40	1.03	1.93	0.00	0.00	2.51	0.00	0.99	3.04	1.89	1.52	2.22	1.64	3.08
502	Sanger	−	−	−	−	−	+	−	−	−	−	−	−	−	−	−	−
	NGS (%)	1.10	0.00	1.60	0.00	0.00	80.97	0.00	8.45	0.00	1.31	1.17	1.35	1.49	2.27	1.60	0.00
504	Sanger	−	−	−	−	−	−	−	−	−	−	−	−	−	−	−	−
	NGS (%)	0.97	1.89	1.62	1.44	0.00	17.99	0.00	1.22	0.00	0.00	0.94	1.17	1.74	1.22	2.16	0.00
517	Sanger	−	−	−	−	−	−	−	−	−	−	−	−	−	+	−	−
	NGS (%)	0.00	1.56	2.28	0.92	0.00	1.87	0.00	1.36	0.00	1.80	0.00	1.66	1.26	98.85	1.77	0.00
A309	Sanger	−	−	−	−	−	−	−	−	−	−	−	−	−	−	−	+
	NGS (%)	3.12	1.20	2.44	0.00	0.93	0.00	0.00	1.81	1.11	1.47	1.35	2.10	2.86	4.28	2.13	98.31
A320	Sanger	−	−	−	−	−	−	−	−	−	−	−	−	−	−	−	−
	NGS (%)	2.09	1.15	2.59	0.00	6.93	0.00	0.00	1.85	0.96	2.03	2.76	1.98	1.87	1.95	1.68	0.00
A328	Sanger	−	−	−	−	−	−	−	−	−	−	−	−	−	−	−	−
	NGS (%)	2.16	8.32	2.40	0.00	2.05	0.00	0.00	2.54	0.94	1.82	5.45	1.50	2.12	1.58	2.65	0.00

In addition, the frequencies of mutations dectected by NGS were analyzed in patients grouped by HBeAg status, HBV genotype, and liver disease progression. RT sites with significant differences in mutation frequency are shown in Fig. 4A2 to C2. The frequencies of mutations at 15 sites (rt219, rt224, rt237, rt238, rt239, rt241, rt242, rt251, rt255, rt256, rt266, rt270, rt271, rt274, and rt295) were significantly different between HBeAg-positive and HBeAg-negative patients. Significantly higher mutation frequencies were found at rt224 and rt274 in HBeAg-positive patients, while significantly higher mutation frequencies were found at the other 13 sites in HBeAg-negative patients (Fig. 4A2). Moreover, significantly different mutation frequencies at 23 sites (rt202, rt221, rt224, rt227, rt230, rt233, rt235, rt237, rt238, rt239, rt240, rt242, rt243, rt246, rt256, rt267, rt270, rt278, rt279, rt280, rt294, rt332, and rt333) were found between patients infected with genotype B HBV and those infected with genotype C HBV. In patients infected with genotype B HBV, the highest mutation frequencies were at rt202, rt221, rt230, rt233, rt237, rt239, rt240, rt242, rt256, rt270, rt279, rt294, rt332, and rt333, while the highest mutation frequencies were shown at rt224, rt227, rt235, rt238, rt243, rt246, rt267, rt278, and rt280 in those infected with genotype C HBV (Fig. 4B2). Among the HC, CHB, and ALD group, 19 sites with a significant difference in mutation frequency were found, including rt202, rt204, rt205, rt210, rt220, rt233, rt236, rt238, rt239, rt242, rt251, rt266, rt274, rt278, rt280, rt283, rt284, rt286, and rt292. Of those sites mentioned above, mutations at rt202, rt204, rt205, rt210, rt220, rt236, rt238, rt239, rt242, rt278, and rt 292 were most frequent in CHB patients, while mutations at rt233 were most frequent in those in the HC group. Notably, the highest mutation frequencies were found in ALD patients at rt251, rt266, rt274, rt280, rt283, rt284, and rt286 in domain E and the E-RNA H interdomain (Fig. 4C2).

## DISCUSSION

With the widespread use of NAs, there has been increasing numbers of nucleot(s)ide resistance (NA^r^) mutations reported in the HBV RT region. However, the existence of drug resistance mutations in treatment-naive patients remains controversial. In this study, except rtA181T, the classical primary drug resistance mutations (i.e., I169T, A181T/V, T184A/C/F/G/I/L/M/S, A194T, S202C/G/I, M204I/V/S, N236T, and M250I/L/V) were not detected by Sanger sequencing in treatment-naive patients (Table 3), which was consistent with previous reports (6, 25, 26). Considering that Sanger sequencing is a population-based sequencing approach used to detect mutations with an intrahost rate of more than 20% (6, 27), the minor mutations with an intrahost rate of less than 20% were likely to be ignored by Sanger sequencing. In this study, using next-generation sequencing, mutations were found at rt202 (rtS202R/I/N/C/G), rt204 (rtM204L/R/I), rt236 (rtN236T/K/H), and rt250 (rtM250I/L) (Table 5). Although it was reported that naturally occurring NA^r^ mutations were not dominant in the treatment-naive patients, the effect of minor quasispecies with primary NA^r^ mutations on the subsequent antiviral treatment was worth being further investigated.

In the range of amino acid sequences from rt145 to rt289 tested by Sanger sequencing in this study, 12 genotype-dependent amino acid polymorphic positions (rt151, rt191, rt221, rt222, rt223, rt224, rt226, rt238, rt256, rt267, rt269, and rt271) were identified for genotypes B and C; this was important for the definition of genotypic mutation. For example, rtA (alanine) 222T (threonine) was considered to be a novel mutation, according to a previous report (28). However, in this study, alanine and threonine at rt222 were identified to be genotype-dependent wild-type amino acids for genotypes B and C, respectively. Moreover, a previous document showed that in the polymorphic positions from rt145 to rt289 which were analyzed in this study, rt221, rt224, rt238, and rt256 were identified to be the genotype-dependent amino acid polymorphic positions for genotypes B and C in patients from Beijing, China (6). Compared with this result described above, besides rt221, rt224, rt238, and rt256, another 8 amino acid positions (rt151, rt191, rt222, rt223, rt226, rt267, rt269, and rt271) were identified as being polymorphic sites in patients from Fujian, China. The difference implied that even in different areas of the same country, there were also some differences in the HBV wild-type sequence. Therefore, instead of using the same reference sequence in different areas, it might be more appropriate for genotypic mutation analysis that the consensus sequence was obtained from the alignment for HBV nucleotide sequences among local treatment-naive patients and regarded as the reference sequence.

Currently, the well-known classical NA resistance mutations are mainly located in domains B, C, D, and E, such as rtI169T, rtA181T/V, and rtT184A/C/F/G/I/L/M (located in domain B), rtS202C/G/I and rtM204I/V/S (located in domain C), rtN236T (located in domain D), and rtM250I/L/V (located in domain E). Therefore, mutations in these domains have a greater risk of NA resistance. A previous study had shown that the prevalence of mutations in the A-B interdomain was higher than in the RT domain or non-A-B interdomains (6). However, the difference in prevalence of mutations between each other of sections in RT was still unknown. Therefore, we have analyzed a total of 46 mutations in domain B, the B-C interdomain, domain C, the C-D interdomain, domain D, the D-E interdomain, and domain E from rt164 to rt270 to address that issue and assess the risk of NA resistance. The results showed that mutant hot spot fractions were different among the HC, CHB, and ALD groups. Given that some patients in the CHB group might be eligible for antiviral treatment, mutations in domain E with significantly higher mutation frequency in the CHB group (Fig. 1), such as rtS/C256G, which is associated with ETV treatment, should be noticed (29).

In this study, we compared the clinical characteristics of patients with and without RT mutations. We found that people with RT mutations, especially with multiple mutations, were apparently older than those without mutations and had a significantly lower positive ratio of HBeAg, lower HBV DNA load, and lower HBsAg level (Table 4). Recently, it was documented that HBV seemed to be under host immune pressure in chronic HBV infection, particularly in the HBeAg-negative status (30). Therefore, in this study, patients with RT mutations who had lower positive ratios of HBeAg might be under greater immune pressure. In turn, the greater immune pressure could result in the decreased HBV DNA load and HBsAg level. Also, under the greater immune pressure, more mutations might be selected for and accumulate for immune escape over time. In fact, the results in this study have shown that many mutations in RT sites (36/56 [64.29%]) were found to change the B or T cell epitopes in the RT or S protein (Table 3). Interestingly, although many mutations could change the epitopes, mutations found in the D-E interdomain and domain E appeared not to change the B or T cell epitopes in the RT or S protein, implying that mutations in the D-E interdomain and domain E were not responsible for immune escape. In addition, the correlation between RT mutations and stage of liver fibrosis was analyzed in this study. The results have shown that patients with multiple RT mutations have more severe liver fibrosis than do those without mutations (Fig. 2), implying that mutations in RT might be involved in the progression of liver disease. However, the mechanism of the relationship between multiple RT mutations and liver fibrosis was still unclear.

Further, next-generation sequencing was performed on 66 treatment-naive patients, and Sanger sequencing was also performed on 63 of those patients at the same time. We found that more mutations occurred in HBeAg-negative patients, genotype B-infected patients, and CHB patients than in HBeAg-positive patients, genotype C-infected patients, and HC or ALD patients, respectively (Fig. 3A and 4A1 to C1), which might be attributed to the higher immune pressure. Indeed, Desmond et al. (30) defined viral adaptation as the presence of the relevant HLA and the escaped amino acid, and they found that the adaptation was significantly lower in HBeAg-positive than in HBeAg-negative patients among genotype B-infected patients, while this result was not significant among those infected with genotype C HBV. Furthermore, it should be noticed that more mutations occurred at rt251, rt266, rt274, rt280, rt283, rt284, and rt286 located in domain E and the E-RNA H interdomain than in the other RT sections in ALD patients (Fig. 4C2), indicating that those mutations were associated with liver disease progression. However, the phenotypic mechanisms of those mutations remain unclear and deserve to be elucidated.

In conclusion, through Sanger sequencing and NGS, the present study realized the analysis of mutations in the RT region in different liver disease stages, providing a possible explanation to illuminate the mechanisms of their evolution and selection under varied levels of immune pressure. Classical primary drug resistance mutations were found at a low rate by next-generation sequencing in the treatment-naive patients but were not detected by Sanger sequencing. It was worth investigating the effect of those NA^r^ mutations in a low rate on the subsequent treatment. In addition, the correlation was also discussed between the accumulation of RT mutations and clinical indexes, including HBeAg, HBV DNA, HBsAg, age, and stage of liver fibrosis, emphasizing the clinical significance of the overall effect of naturally occurring RT mutations on the treatment-naive patients. The detailed functional mechanisms of particular mutations need to be further explored.

## References

[1] Shan S, Cui F, Jia J 2018 How to control highly endemic hepatitis B in Asia. Liver Int 38(Suppl 1):122–125. doi:10.1111/liv.13625. 29427490

[2] Lumley SF, McNaughton AL, Klenerman P, Lythgoe KA, Matthews PC 2018 Hepatitis B virus adaptation to the CD8^+^ T cell response: consequences for host and pathogen. Front Immunol 9:1561. doi:10.3389/fimmu.2018.01561. 30061882 PMC6054973

[3] European Association for the Study of the Liver. 2017 EASL 2017 clinical practice guidelines on the management of hepatitis B virus infection. J Hepatol 67:370–398. doi:10.1016/j.jhep.2017.03.021. 28427875

[4] Pacheco SR, Dos Santos M, Stocker A, Zarife MAS, Schinoni MI, Parana R, Dos Reis MG, Silva LK 2017 Genotyping of HBV and tracking of resistance mutations in treatment-naive patients with chronic hepatitis B. Infect Drug Resist 10:201–207. doi:10.2147/IDR.S135420. 28740410 PMC5503499

[5] Zhu B, Wang T, Wei X, Zhuo Y, Liu A, Zhang G 2017 Accumulation of mutations in reverse transcriptase of hepatitis B virus is associated with liver disease severity in treatment-naive Chinese patients with chronic hepatitis B. Adv Clin Exp Med 26:1123–1129. doi:10.17219/acem/63998. 29211361

[6] Liu BM, Li T, Xu J, Li XG, Dong JP, Yan P, Yang JX, Yan L, Gao ZY, Li WP, Sun XW, Wang YH, Jiao XJ, Hou CS, Zhuang H 2010 Characterization of potential antiviral resistance mutations in hepatitis B virus reverse transcriptase sequences in treatment-naive Chinese patients. Antiviral Res 85:512–519. doi:10.1016/j.antiviral.2009.12.006. 20034521

[7] Singla B, Chakraborti A, Sharma BK, Kapil S, Chawla YK, Arora SK, Das A, Dhiman RK, Duseja A 2013 Hepatitis B virus reverse transcriptase mutations in treatment naive chronic hepatitis B patients. J Med Virol 85:1155–1162. doi:10.1002/jmv.23608. 23918533

[8] Moehlen M, De Medina M, Hill M, Jeffers L, Schiff ER, Martin P 2013 Absence of hepatitis B resistance mutants before introduction of oral antiviral therapy. ISRN Hepatol 2013:130384. doi:10.1155/2013/130384. 27335823 PMC4890864

[9] Xu J, Wu B, Wang J-H, Huang L, Wang D-y, Zhao L, Zhao G-p, Wang Y 2015 Pre-existing mutations in reverse transcriptase of hepatitis B virus in treatment-naive Chinese patients with chronic hepatitis B. PLoS One 10:e0117429. doi:10.1371/journal.pone.0117429. 25821965 PMC4379075

[10] Zhao Y, Wu J, Sun L, Liu G, Li B, Zheng Y, Li X, Tao J 2016 Prevalence of mutations in HBV DNA polymerase gene associated with nucleos(t)ide resistance in treatment-naive patients with chronic hepatitis B in central China. Braz J Infect Dis 20:173–178. doi:10.1016/j.bjid.2015.12.006. 26876337 PMC9427582

[11] Kim JE, Lee SY, Kim H, Kim KJ, Choe WH, Kim BJ 2017 Naturally occurring mutations in the reverse transcriptase region of hepatitis B virus polymerase from treatment-naive Korean patients infected with genotype C2. World J Gastroenterol 23:4222–4232. doi:10.3748/wjg.v23.i23.4222. 28694662 PMC5483496

[12] Yamani LN, Yano Y, Utsumi T, Wasityastuti W, Rinonce HT, Widasari DI, Juniastuti Lusida MI, Soetjipto, Hayashi Y 2017 Profile of mutations in the reverse transcriptase and overlapping surface genes of hepatitis B virus (HBV) in treatment-naïve Indonesian HBV carriers. Jpn J Infect Dis 70:647–655. doi:10.7883/yoken.JJID.2017.078. 29093313

[13] Qian F, Zou W, Qin J, Li D 2017 Naturally occurring genotypic drug-resistant mutations of HBV in Huzhou, China: a single-center study. Infect Drug Resist 10:507–509. doi:10.2147/IDR.S149992. 29276396 PMC5733919

[14] Yang JH, Zhang H, Chen XB, Chen G, Wang X 2013 Relationship between hepatocellular carcinoma and hepatitis B virus genotype with spontaneous YMDD mutations. World J Gastroenterol 19:3861–3865. doi:10.3748/wjg.v19.i24.3861. 23840126 PMC3699046

[15] Shen T, Yan XM 2014 Hepatitis B virus genetic mutations and evolution in liver diseases. World J Gastroenterol 20:5435–5441. doi:10.3748/wjg.v20.i18.5435. 24833874 PMC4017059

[16] Tatsukawa M, Takaki A, Shiraha H, Koike K, Iwasaki Y, Kobashi H, Fujioka S, Sakaguchi K, Yamamoto K 2011 Hepatitis B virus core promoter mutations G1613A and C1653T are significantly associated with hepatocellular carcinoma in genotype C HBV-infected patients. BMC Cancer 11:458. doi:10.1186/1471-2407-11-458. 22014121 PMC3214198

[17] Wei F, Zheng Q, Li M, Wu M 2017 The association between hepatitis B mutants and hepatocellular carcinoma: a meta-analysis. Medicine (Baltimore, MD) 96:e6835. doi:10.1097/MD.0000000000006835. PMC5428601 28489767

[18] Yang G, Liu Z, Yang J, Luo K, Xu Y, He H, Fu Q, Yu S, Wang Z 2017 Quasispecies characteristics in mother-to-child transmission of hepatitis B virus by next-generation sequencing. J Infect 75:48–58. doi:10.1016/j.jinf.2017.04.012. 28483405

[19] Borroto-Esoda K, Miller MD, Arterburn S 2007 Pooled analysis of amino acid changes in the HBV polymerase in patients from four major adefovir dipivoxil clinical trials. J Hepatol 47:492–498. doi:10.1016/j.jhep.2007.06.011. 17692425

[20] Stuyver LJ, Locarnini SA, Lok A, Richman DD, Carman WF, Dienstag JL, Schinazi RF 2001 Nomenclature for antiviral-resistant human hepatitis B virus mutations in the polymerase region. Hepatology 33:751–757. doi:10.1053/jhep.2001.22166. 11230757

[21] Shi Y, Wang J, Wang Y, Wang A, Guo H, Wei F, Mehta SR, Espitia S, Smith DM, Liu L, Zhang Y, Chen D 2016 A novel mutant 10Ala/Arg together with mutant 144Ser/Arg of hepatitis B virus X protein involved in hepatitis B virus-related hepatocarcinogenesis in HepG2 cell lines. Cancer Lett 371:285–291. doi:10.1016/j.canlet.2015.12.008. 26706415 PMC4934374

[22] Choi YM, Lee SY, Kim BJ 2018 Naturally occurring hepatitis B virus reverse transcriptase mutations related to potential antiviral drug resistance and liver disease progression. World J Gastroenterol 24:1708–1724. doi:10.3748/wjg.v24.i16.1708. 29713126 PMC5922991

[23] Desmond CP, Bartholomeusz A, Gaudieri S, Revill PA, Lewin SR 2008 A systematic review of T-cell epitopes in hepatitis B virus: identification, genotypic variation and relevance to antiviral therapeutics. Antivir Ther 13:161–175. 18505168

[24] Lin YM, Jow GM, Mu SC, Chen BF 2013 Naturally occurring hepatitis B virus B-cell and T-cell epitope mutants in hepatitis B vaccinated children. ScientificWorldJournal 2013:571875. doi:10.1155/2013/571875. 24379746 PMC3860134

[25] Pollicino T, Maimone S, Isgrò G, Brancatelli S, Raffa G, Caccamo G, Squadrito G, Raimondo G 2007 Variability of the HBV pol gene reverse-transcriptase domain in viral isolates from untreated and lamivudine-resistant chronic hepatitis B patients. Dig Liver Dis 39:A7. doi:10.1016/j.dld.2006.12.037.

[26] Nguyen MH, Trinh HN, Garcia RT, Nguyen LH, Vutien P, Ha NB, Nguyen HA, Nguyen KK, Keeffe EB, Ha NB 2008 Prevalence of HBV DNA polymerase (B-DNA Pol) mutations in 345 patients with treatment-naïve chronic hepatitis B (CHB). Gastroenterology 134:A-310. doi:10.1016/S0016-5085(08)61445-6.

[27] Liu C, Lin J, Chen H, Shang H, Jiang L, Chen J, Ye Y, Yang B, Ou Q 2014 Detection of hepatitis B virus genotypic resistance mutations by coamplification at lower denaturation temperature-PCR coupled with Sanger sequencing. J Clin Microbiol 52:2933–2939. doi:10.1128/JCM.01127-14. 24899029 PMC4136161

[28] Wei C, Chong YT, Wen JZ, Li YW, Li G 2011 Characterization of hepatitis virus B isolated from a multi-drug refractory patient. Virus Res 155:254–258. doi:10.1016/j.virusres.2010.10.018. 20970466

[29] Colonno RJ, Rose R, Baldick CJ, Levine S, Pokornowski K, Yu CF, Walsh A, Fang J, Hsu M, Mazzucco C, Eggers B, Zhang S, Plym M, Klesczewski K, Tenney DJ 2006 Entecavir resistance is rare in nucleoside naive patients with hepatitis B. Hepatology 44:1656–1665. doi:10.1002/hep.21422. 17133475

[30] Desmond CP, Gaudieri S, James IR, Pfafferott K, Chopra A, Lau GK, Audsley J, Day C, Chivers S, Gordon A, Revill PA, Bowden S, Ayres A, Desmond PV, Thompson AJ, Roberts SK, Locarnini SA, Mallal SA, Lewin SR 2012 Viral adaptation to host immune responses occurs in chronic hepatitis B virus (HBV) infection, and adaptation is greatest in HBV e antigen-negative disease. J Virol 86:1181–1192. doi:10.1128/JVI.05308-11. 22072755 PMC3255822

[31] Hou J, Wang G, Wang F, Cheng J, Ren H, Zhuang H, Sun J, Li L, Li J, Meng Q, Zhao J, Duan Z, Jia J, Tang H, Sheng J, Peng J, Lu F, Xie Q, Wei L, Chinese Society of Hepatology, Chinese Medical Association, Chinese Society of Infectious Diseases, Chinese Medical Association 2017 Guideline of prevention and treatment for chronic hepatitis B (2015 update). J Clin Transl Hepatol 5:297–318. 29226097 10.14218/JCTH.2016.00019 PMC5719188

